# Systematic evaluation of gene variants linked to hearing loss based on allele frequency threshold and filtering allele frequency

**DOI:** 10.1038/s41598-019-41068-6

**Published:** 2019-03-14

**Authors:** John Hoon Rim, Joon Suk Lee, Jinsei Jung, Ji Hyun Lee, Seung-Tae Lee, Jong Rak Choi, Jae Young Choi, Min Goo Lee, Heon Yung Gee

**Affiliations:** 1 Department of Pharmacology, Brain Korea 21 PLUS Project for Medical Sciences, Yonsei University College of Medicine, Seoul, 03722 Korea; 2 Department of Medicine, Physician-Scientist Program, Yonsei University Graduate School of Medicine, Seoul, 03722 Korea; 3 Department of Laboratory Medicine, Yonsei University College of Medicine, Seoul, 03722 Korea; 4 Department of Otorhinolaryngology, Brain Korea 21 PLUS Project for Medical Sciences, Yonsei University College of Medicine, Seoul, 03722 Korea; 5 Department of Dermatology, Seoul St. Mary’s Hospital, Brain Korea 21 PLUS Project for Medical Sciences, College of Medicine, The Catholic University of Korea, Seoul, Republic of Korea

## Abstract

As the number of genes identified for linkage to hearing loss has been increasing and more public databases have become available, we aimed to systematically evaluate all variants reported for nonsyndromic hearing loss (NSHL) based on their allele frequencies (AFs) in the general population. Among the 3,549 variants in 97 NSHL genes reported as pathogenic/likely pathogenic in ClinVar and HGMD, 1,618 were found in public databases (gnomAD, ExAC, EVS, and 1000G). To evaluate the pathogenicity of these variants, we employed AF thresholds and NSHL-optimized ACMG guidelines. AF thresholds were determined using a high-resolution variant frequency framework and Hardy-Weinberg equilibrium calculation: 0.6% and 0.1% for recessive and dominant genes, respectively. Filtering AFs of variants linked to NSHL were obtained based on AFs reported in gnomAD and ExAC. We found that 48 variants in 23 genes had filtering AFs above the suggested thresholds and assumed that these variants might be benign based on their filtering AFs. 47 variants, except for one notorious high-frequency *GJB2* mutation (c.109G > A; p.Val37Ile), were confirmed to be benign/likely benign by the NSHL-optimized ACMG guidelines. The proposed systematic approach will aid in precise evaluation of NSHL variant pathogenicity in the context of filtering AFs, AF thresholds, and NSHL-specific ACMG guidelines, thus improving NSHL diagnostics.

## Introduction

Rapid advancements in sequencing technologies and bioinformatic tools for genomic data analysis have enabled clinicians and researchers to take a step forward in the implementation of precision medicine for Mendelian disorders. Large population databases and publicly accessible repositories of disease-causing mutations facilitate clinical interpretation of gene variants identified by next-generation sequencing (NGS) in individuals with genetic disorders^1^. In addition, functional assessment of variants both *in vitro* and *in vivo* is important for determining their contribution to disease pathogenesis. Clinical application of NGS made it possible to identify diagnostic and prognostic signatures in a number of diseases, including hereditary hearing loss, whose genetic landscape has been actively explored from both diagnostic and therapeutic aspects.

Hearing loss is the most common sensory disorder that affects approximately one in every 500 newborns worldwide^2^. Over two-thirds of hereditary hearing loss cases are diagnosed as nonsyndromic hearing loss (NSHL), in which the hearing loss phenotype is the only feature observed without additional symptoms^3^. Since 2015, application of NGS technology to discovering novel genetic causes of NSHL enabled identification of 16 additional genes, whose variants have been linked to NSHL development, and currently, approximately a hundred genes are implicated in the disease. However, genetic heterogeneity and phenotypic variability of NSHL makes precise interpretation of variants identified by NGS a challenge. Furthermore, it is technically difficult to examine the pathogenic impact of some gene variants associated with NSHL, such as those of *MYO15A* and *CDH23*, because of very large gene sizes and the absence of relevant functional tests *in vitro*.

To improve the clinical utility of NGS in NSHL, one useful approach is to consider the allele frequency (AF) of a gene variant. Large reference datasets such as Exome Aggregation Consortium (ExAC) and Genome Aggregation Database (gnomAD) not only provide high-resolution variant frequencies, but also allow filtering AFs with robust statistical significance^4^. In addition, disease-specific application of optimized American College of Medical Genetics (ACMG) guidelines has been attempted for various genetic disorders based on the importance of pathomechanistic diversity^5^. Furthermore, widespread availability of mutation databases such as ClinVar and Human Gene Mutation Database (HGMD) encouraged researchers to refine strategies for gene variant interpretation and even to reassess outdated mutations reported before the advent of high-precision genetic tools and large-scale databases^6–8^.

In this study, we aimed to systemically evaluate publicly reported genetic variants associated with NSHL in terms of their pathogenicity by applying thresholds of AFs newly calculated for the general population. The new classification results were validated using the NSHL-specific ACMG guidelines and compared with previous reports.

## Methods

### Systematic collection of pathogenic variants for curated NSHL genes

We comprehensively evaluated the evidential level of cause-and-effect relationship for the genes associated with NSHL and created a final list of 97 causative genes that were reported in all three examined databases: the Hereditary Hearing Loss (http://hereditaryhearingloss.org/), Deafness Variation Database (http://deafnessvariationdatabase.org/), and Online Mendelian Inheritance in Man (OMIM) database. Furthermore, we selected genes that have enough evidence of association with NSHL, such as those with evidential level 2–3 according to previous studies^9–11^ and with more than three clinical reports of hearing loss patients carrying gene variants (Supplementary Table S1). Next, we selected all reported gene variants classified as presumably pathogenic in HGMD Professional (accessed May, 2017) or ClinVar (20170501.ver) databases; variants annotated as “DM” or “DM?” in HGMD or as “pathogenic” or “likely pathogenic” in ClinVar were compiled. The annotation and nomenclature of the variants were confirmed using the Mutalyzer Name Checker tool based on clinically relevant transcripts in each gene.

### General population datasets

To maximize the volume of general population data, we utilized four widely used control databases: (1) gnomAD (n = 141,456, http://gnomad.broadinstitute.org/), (2) ExAC (n = 60,706, http://exac.broadinstitute.org/), (3) NHLBI Exome Sequencing Project (n = 6,503, http://evs.gs.washington.edu/EVS/), and (4) 1000 Genomes Project Phase 3 database (1000 G; n = 2,504, http://www.internationalgenome.org/). We used ‘observed AFs’ representing the count ratio of the actually detected minor alleles to reliably sequenced alleles. All reportedly pathogenic or likely pathogenic variants of the 97 NSHL-linked genes were searched for observed AF separately in the four databases, which have distinct demographic composition in terms of ethnicity and population size. All gnomAD and ExAC data were checked using the “pass” filter to include only variants with appropriate coverage.

### Determination of AF thresholds

As the AF of a variant in the general population is an essential criterion for pathogenicity interpretation, various approaches were used to define the AF threshold at which a variant could be interpreted as too common to be classified as “definitely pathogenic”. Since AF thresholds might be fairly different depending on disease nature, we applied empirical approaches as well as theoretical calculations to determine appropriate AF thresholds for NSHL variants.

First, empirical bottom-up analysis using AF thresholds of 0.005%, 0.01%, 0.05%, and 0.1% was adopted from a previous study^12^ to evaluate overall AF distributions of NSHL-linked gene variants reported to be pathogenic in HGMD or ClinVar. As different AF thresholds are used in different studies, we chose the 0.05% threshold suggested in a previous report^13^ and, in addition, applied two-fold threshold values.

Second, theoretical calculations were performed to obtain evidence-based AF thresholds. Different approaches were utilized depending on the mode of inheritance. For dominant genes, the AF cut-off value was obtained using the Hardy-Weinberg equilibrium based on the prevalence of hereditary NSHL, since no single mutation was reported to represent the majority of dominant NSHL. For recessive genes, we applied the high-resolution variant frequency framework suggested by Whiffin *et al*.^14^.

### Calculation of filtering AF using the gnomAD and ExAC datasets

Filtering AF was previously defined as the threshold disease-specific “maximum credible AF” at or below which the disease could not plausibly be caused by that variant^14^. Filtering AFs were computed using the “inverse AF” calculator of a web-based tool (http://cardiodb.org/allelefrequencyapp/). We calculated filtering AFs using observed AFs across all ethnicities in the gnomAD and ExAC datasets rather than AFs for sub-populations.

### ACMG guideline application and NSHL-specific rules

To systematically evaluate the pathogenic potential of presumably pathogenic variants with AFs higher than the thresholds defined for the general population, we applied the 2015 ACMG guidelines for variant classification with InterVar, one of the most commonly used bioinformatics tools for clinical interpretation of genetic variants^15^. To enhance the accuracy of analysis, we optimized the ACMG guidelines for NSHL based on updates of the NSHL genetic background during the last decade. Detailed parameterization of each ACMG guideline components optimized for NSHL is explained in Supplementary Notes. Briefly, filtering AF was used for analysis of a population database; in addition, the pLI score defined as the probability of a gene being intolerant to a loss-of-function (LoF) mutation^9^ and guidelines for LoF prediction^16^ were used to determine LoF gene variants linked to NSHL pathophysiology. Results of reliable functional studies on hearing loss were manually curated on the evidential basis.

### Comparison of in-silico prediction results between rare and common missense variants according to filtering AF

To analyze the association of missense mutations with the scarcity of variants and mode of inheritance, we applied three most widely used algorithms: PolyPhen-2 (PP2), Sorting Intolerant from Tolerant (SIFT), and Consensus deleteriousness of non-synonymous single nucleotide variants (Condel)^17–19^. The Mann-Whitney test was used to evaluate statistical significance of differences between common and rare variants, and a *P* value less than 0.05 was considered significant.

## Results

### NSHL-related variants pooled from publicly available databases

**Figure 1:**
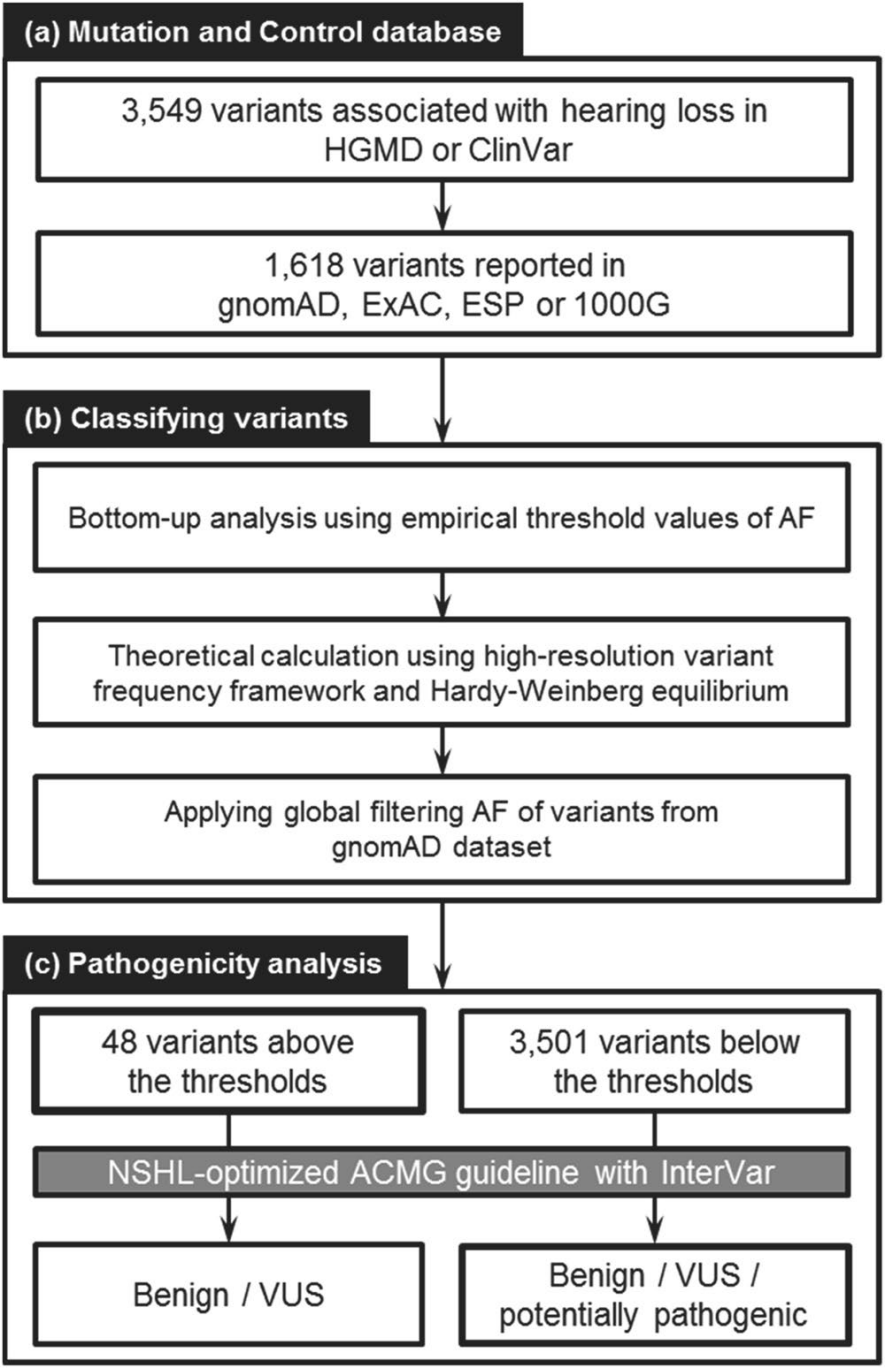
Overall workflow of the study. (**a**) Deafness variants reported in public mutation databases. The number of variants deposited in the Human Gene Mutation Database (HGMD) and ClinVar were 3,082 and 1,210, respectively. Variants reported as likely pathogenic or pathogenic in HGMD or ClinVar were further examined. Among the 3,549 variants, 1,618 were reported in gnomAD, ExAC, EVS, and 1000 G control datasets. (**b**) Variant classification according to allele frequency (AF). Bottom-up analysis, theoretical calculation and filtering AF were applied. (**c**) Evaluation of variant pathogenicity. Variants were interpreted according to the NSHL-optimized ACMG guidelines. VUS, variants of unknown significance.

For the 97 NSHL causative genes curated from three databases (Hereditary Hearing Loss, Deafness Variation Database, and OMIM), a total of 3,549 variants were reported as presumably pathogenic either in the HGMD or ClinVar. Among them, 1,618 (45.6%) were present in at least one of four control datasets: gnomAD, ExAC, EVS, and 1000G (Fig. 1).

### AF threshold values determined by two approaches

#### Bottom-up analysis

An empirical approach using different AF threshold values (0.005%, 0.01%, 0.05%, and 0.1%) to examine the rarity of a variant in large population datasets revealed that 1,598 out of 3,549 (45.0%) variants were reported in gnomAD, whereas only 367 (10.3%) variants were in 1000G (Supplementary Table S2), demonstrating the higher resolution from more number of sequenced individuals. In addition, 1,110 (31.3%) and 733 (20.7%) variants with AFs of 0 < AF < 0.005% were present in gnomAD and ExAC, respectively, showing the power of sample size (141,456 in gnomAD vs. 60,706 in ExAC) (Supplementary Table S2). Furthermore, no variant had AF of 0 < AF < 0.005% based on EVS or 1000G; however, this was due to small sample sizes of EVS or 1000G (Supplementary Table S2). Nevertheless, the numbers of variants with AF of less than 0.005% were similar regardless of databases (Supplementary Table S2).

#### Theoretical calculations using the Hardy-Weinberg equilibrium and maximum credible AF

For dominant genes, the AF threshold of 0.1% was obtained through Hardy-Weinberg equilibrium (see Supplementary Notes)^20^. For recessive genes, the AF threshold was determined using a theoretical formula proposed by Whiffin *et al*.^20^, which considers reliable estimates for NSHL prevalence, penetrance, and allelic contribution. Using the *GJB2* variant (c.35delG; p.Gly12Valfs*2), which is the most prevalent recessive mutation according to Sloan-Heggen *et al*.^21^, the AF threshold was determined as:

√(0.002 × 0.8 × 0.7) × 0.3789 × √(0.2159 × 1) = 0.6% (see Supplementary Notes).

### Reclassification of previously reported pathogenic NSHL variants using observed and filtering AFs

**Figure 2:**
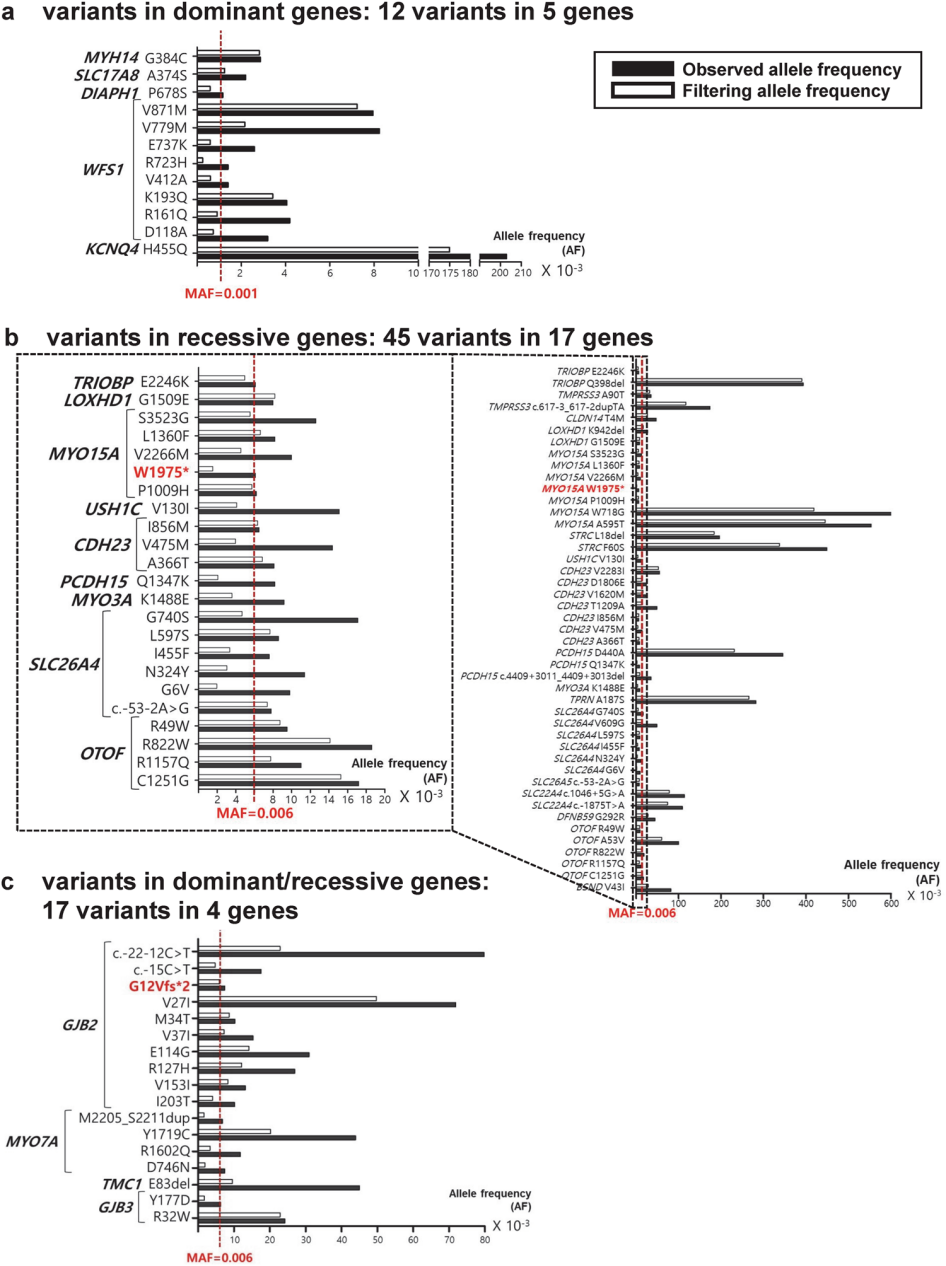
Comparison of observed and gnomAD filtering allele frequencies (AFs) for variants with observed AFs higher than AF thresholds. (**a**) Twelve variants in 5 dominant genes presented observed AFs over 0.1%; among them, 6 variants were reclassified as rare with filtering AFs lower than 0.1% (red vertical dashed line). (**b**) Forty five variants in 17 recessive genes presented observed AFs over 0.6%; among them, 13 variants were reclassified as rare with filtering AFs lower than 0.6% (red vertical dashed line in the magnified plot). In particular, one nonsense variant of the *MYO15A* gene (c.5925G > A, p.Trp1975*; red bolded variant), which was reclassified as rare based on filtering AF, was classified as pathogenic according to ACMG guideline interpretation. Among 45 variants, 23 variants with both observed and filtering AFs lower than 2.0% are magnified in the subset for clarity. (**c**) Seventeen variants in 4 dominant/recessive genes presented observed AF over 0.6%; among them, 7 variants were reclassified as rare with filtering AFs lower than 0.6% (red vertical dashed line). In particular, one frameshift variant of the *GJB2* gene (c.35delG, p.Gly12Valfs*2; red bolded variant), which was reclassified as rare variant based on filtering AF, was classified as pathogenic according to ACMG guideline interpretation.

To examine the validity of AF thresholds derived from theoretical calculations, we investigated the distribution of variants according to their observed AFs as well as filtering AFs. For dominant genes, 12 variants in 5 genes showed observed AFs over our threshold of 0.1%. However, when filtering AFs of these 12 variants were applied for reassessment, only 6 (50%) remained as common variants with AFs over 0.1% (Fig. 2a). For recessive genes, 45 variants in 17 genes presented observed AFs over 0.6%; however, 28.9% (13/45) of them were reclassified to the category of rare variants with filtering AFs lower than the threshold (0.6%) (Fig. 2b). For genes which exhibit both dominant and recessive patterns, 41.2% (7/17) of variants with observed AFs over 0.6% were classified as rare based on filtering AFs lower than 0.6% (Fig. 2c).

### Evaluation of pathogenicity of reclassified NSHL variants using the ACMG guidelines and NSHL-specific rules

We applied the NSHL-specific ACMG guidelines to evaluate pathogenicity of variants previously considered as common and reclassified as rare using filtering AF and our thresholds (Table 1). Among a total of 26 variants in 15 genes that fulfilled the “PM2” component (i.e., absent from controls or observed at an extremely low frequency if recessive) for a sufficiently low filtering AF under our thresholds, two variants had already been classified as pathogenic even without the addition of the “PM2” component. The numbers of individuals with homozygous variants in the gnomAD database ranged from 0 to 36 and recessive genes had more numbers of homozygotes than dominant genes (Table 1). When we also applied recently released the Expert Specified ACMG guidelines for genetic hearing loss developed by ClinGen Working Group^13^ and updated Deafness Variation Database (DVD v8.2)^22^, the data presented high concordance with our results for all the variants (Table 1). On the other hand, almost all of the 48 variants (except for *GJB2* c.109G > A; p.Val37Ile), which had both observed and filtering AFs higher than the thresholds, were classified as benign or likely benign (Supplementary Table S3).

**Table 1:** Profiles of variants with observed AF above thresholds and filtering gnomAD AF below thresholds.

Gene Symbol	Nucleotide Change	Amino acid Change	dbSNP	Observed AF (%)	Contributing Dataset	Filtering AF (%, gnomAD)	Filtering AF (%, ExAC)	Homozygote in gnomAD	HGMD	ClinVar	NSHL-optimized ACMG class	Expert specified ACMG class by ClinGen^13^	DVD classifi-cation
**DOMINANT GENES** (AF threshold: 0.1%)													
*WFS1*	c.353A > C	p.Asp118Ala	rs71524349	0.3195	1000G	0.0726	0.0892	0	DM	LB	VUS	Likely benign*	Benign
*WFS1*	c.482G > A	p.Arg161Gln	rs115346085	0.4193	1000G	0.0905	0.0820	2	DM?	—	Likely benign	Likely benign*	Benign
*WFS1*	c.1235 T > C	p.Val412Ala	rs144951440	0.1398	1000G	0.0600	0.0723	1	DM	LB	Likely benign	Likely benign*	Benign
*WFS1*	c.2195G > A	p.Arg732His	rs149013740	0.1398	1000G	0.0251	0.0278	0	—	VUS|LP	VUS	Likely benign*	Benign
*WFS1*	c.2209G > A	p.Glu737Lys	rs147834269	0.2596	1000G	0.0592	0.0782	3	DM	LB	Likely benign	Likely benign*	Benign
*DIAPH1*	c.2032C > T	p.Pro678Ser	rs186370335	0.1166	ExAC	0.0586	0.0773	0	DM?	LB	Likely benign	Likely benign*	Benign
**RECESSIVE GENES** (AF threshold: 0.6%)													
*SLC26A4*	c.17G > T	p.Gly6Val	rs111033423	0.9800	ExAC	0.1964	0.8686	6	DM?	B|LB	Benign	Likely benign**	Benign
*SLC26A4*	c.970A > T	p.Asn324Tyr	rs36039758	1.1382	1000G	0.3040	0.2850	10	DM?	B|LB	Benign	Likely benign**	Benign
*SLC26A4*	c.1363A > T	p.Ile455Phe	rs375576481	0.7588	1000G	0.3361	0.4050	22	DM?	B	Benign	Likely benign**	Benign
*SLC26A4*	c.2218G > A	p.Gly740Ser	rs17154353	1.7100	ESP	0.4678	0.4070	29	DM?	B|LB	Likely benign	Benign	Benign
*MYO3A*	c.4462A >G	p.Lys1488Glu	rs34204285	0.9185	1000G	0.3590	0.3530	15	DM	VUS|B	Likely benign	Likely benign*	Benign
*PCDH15*	c.4039C > A	p.Gln1347Lys	rs61731387	0.8187	1000G	0.2066	0.2054	8	DM?	—	Likely benign	Likely benign*	Benign
*CDH23*	c.1423G > A	p.Val475Met	rs62622410	1.4377	1000G	0.3967	0.3530	25	DM?	—	Likely benign	Likely benign**	Benign
*USH1C*	c.388G > A	p.Val130Ile	rs55843567	1.5093	ESP	0.4109	0.3780	23	DM?	VUS|B	Likely benign	Likely benign*	Benign
*MYO15A*	c.3026C > A	p.Pro1009His	rs117612144	0.6245	ExAC	0.5727	0.5872	27	DM	B	Likely benign	Benign*	Benign
*MYO15A*	c.5925G > A	p.Trp1975Ter	rs375290498	0.6142	ExAC	0.1528	0.5311	2	DM	VUS|P	**Pathogenic**	Likely Pathogenic*	**Likely pathogenic**
*MYO15A*	c.6796G > A	p.Val2266Met	rs114274755	0.9984	1000G	0.4541	0.7514	5	DM?	B|LB	Likely benign	Likely benign*	Benign
*MYO15A*	c.10573A >G	p.Ser3525Gly	rs182332665	1.2579	1000G	0.5530	0.6025	35	DM?	B	Likely benign	Likely benign*	Benign
*TRIOBP*	c.6736G > A	p.Glu2246Lys	rs138139146	0.6120	ExAC	0.4966	0.5700	3	DM	—	Likely benign	Benign*	Benign
**DOMINANT/RECESSIVE GENES** (AF threshold: 0.6%)													
*GJB3*	c.529T >G	p.Tyr177Asp	rs80297119	0.6305	ESP	0.1768	0.1422	2	DM?	B|LB	Likely benign	Likely benign*	Benign
*MYO7A*	c.2236G > A	p.Asp746Asn	rs36090425	0.7388	1000G	0.1877	0.2000	7	DM	B	Likely benign	Benign**	Benign
*MYO7A*	c.4805G > A	p.Arg1602Gln	rs139889944	1.1781	1000G	0.3375	0.3117	30	DM?	LB|P	Likely benign	Benign**	Benign
*MYO7A*	c.6614_6634dup	p.Met2205_Ser2211dup	rs563508617	0.6789	1000G	0.1674	0.1806	2	—	—	VUS	Benign**	Benign
*GJB2*	c.608T > C	p.Ile203Thr	rs76838169	1.0184	1000G	0.4016	0.3955	31	DM?	B	Benign	Benign**	Benign
*GJB2*	c.35delG	p.Gly12ValfsTer2	rs80338939	0.7429	ESP	0.5946	0.5678	10	DM	P	**Pathogenic**	Pathogenic**	**Pathogenic**
*GJB2*	c.-15C > T	NA	rs72561725	1.7530	ESP	0.004777	0.4414	36	DM?	B|LB	Likely benign	VUS**	Benign

*Corresponding genes were not evaluated by the ClinGen Expert committee; therefore, we applied the modified ACMG guidelines suggested by ClinGen Hearing Loss Working Group.

**Corresponding genes were evaluated by the ClinGen Expert committee but the specific variant was not; therefore, we applied the modified ACMG guidelines suggested by ClinGen Hearing Loss Working Group.

Red bold letters indicate pathogenic mutations concordantly determined by NSHL-optimized ACMG, Expert Specified ACMG by ClinGen, and Deafness Variation Database (DVD) classification, even though their observed AFs were above thresholds and their filtering AFs were below thresholds.

### In-silico prediction analysis of NSHL variants according to AF

In addition, we reviewed the in-silico prediction results for our missense variants based on AFs and gene-specific features. The predicted scores for 245 missense mutations in dominant genes did not show statistically significant differences between variants with AFs below and above the threshold. However, the prediction scores of 1,047 and 668 missense mutations for recessive genes and genes with both inheritance modes, respectively, calculated by three algorithms (PP2, SIFT, and Condel) were statistically different between common and rare variants, i.e., those with AFs above and below the thresholds, respectively (Supplementary Fig. S1).

**Figure 3:**
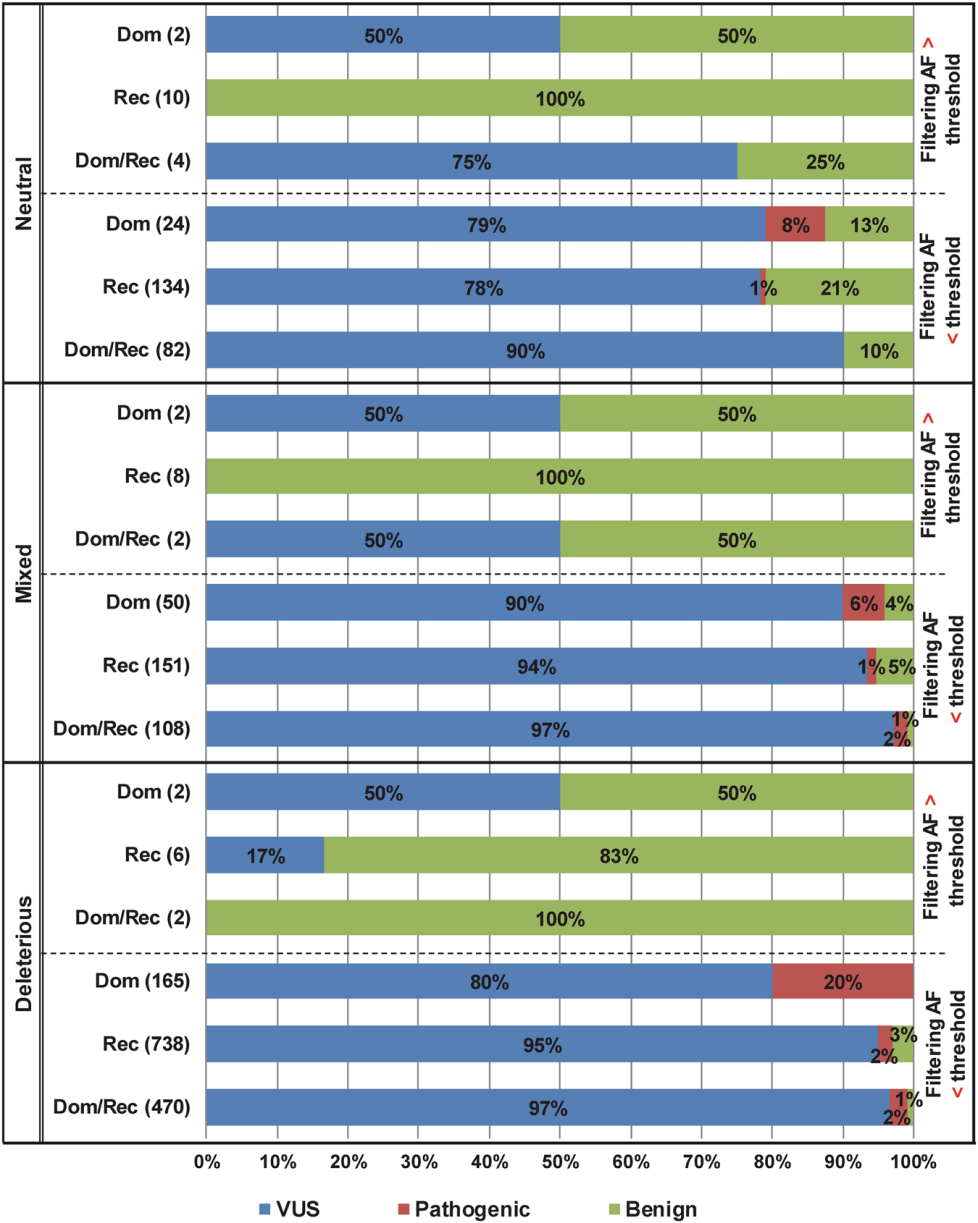
Classification of 1,960 missense variants linked to hearing loss according to in-silico prediction and filtering allele frequency. A total of 1,960 missense mutations were evaluated for pathogenicity using the NSHL-optimized ACMG guidelines and InterVar. Filtering AFs were compared with AF thresholds calculated in this study: 0.1% for dominant genes (Dom), and 0.6% for recessive (Rec) and dominant/recessive (Dom/Rec) genes, respectively. Relative proportions of pathogenic and benign variants and variants of unknown significance (VUS) according to in-silico prediction were shown.

Furthermore, we performed pathogenicity evaluation of 1,960 missense variants according to filtering AFs and InterVar prediction. When an agreement was reached among the three in-silico algorithms in the prediction results, the variants were classified as neutral (all concordant benign results by three algorithms) or deleterious (all concordant damaging results by three algorithms), otherwise mixed (Fig. 3). Interestingly, only pathogenic variants were identified in the rare variant groups with AFs below the thresholds regardless of the prediction consensus among the three algorithms. Although the proportion of pathogenic variants was the highest in the deleterious group (24%), the presence of pathogenic mutations in the neutral and mixed groups was still noticeable (9% in both, respectively).

## Discussion

We systematically evaluated all the reported NSHL-linked pathogenic variants available in population databases by applying clinically plausible AF thresholds, gnomAD and ExAC filtering AF, and NSHL-optimized ACMG guidelines. It should be reassuring for clinicians that over 85% of all presumably pathogenic variants were ultra-rare with observed AFs below 0.05% in bottom-up analysis. However, application of the gnomAD and ExAC filtering AF to 74 reported variants with unexpectedly high observed AFs (i.e., over our AF thresholds) allowed us to safely consider 47 variants with still high filtering AFs as not likely NSHL-related, with the notorious exception of one variant (*GJB2* c.109G > A; p.Val37Ile)^23^. Two pathogenic variants identified by the NSHL-specific ACMG guidelines were among those reclassified from “common” to “rare” by application of the filtering AF, suggesting high clinical utility and accuracy of the AF thresholds determined in this study: 0.1% for dominant genes and 0.6% for recessive genes. In other words, we validated the utility of AF cut-off values using large-scale datasets in the interpretation of NSHL gene variants with undefined rarity.

Several well-designed studies for NSHL variant interpretation have been previously performed. In 2014, Shearer *et al*.^20^ were the first to determine ethnicity-specific AF thresholds for NSHL-linked gene variants based on several population datasets, including EVS and 1000 G. They provided Deafness Variation Database, which is an invaluable resource for researchers and clinicians in the deafness field. However, gnomAD, which comprises more than several million variants and is considered the largest database of human variations, was launched in 2017. Therefore, in this study we combined the recently developed high-resolution framework with the updates in population and mutation datasets, and in the NSHL genetic basis to validate gene variants related to NSHL etiology. As a result, we found that previously suggested AF thresholds (0.05% and 0.5% for autosomal dominant and autosomal recessive genes, respectively), although being fairly satisfactory, were too stringent compared to our thresholds for considering a variant as pathogenic based only on its rarity in population databases. Thus, in Deafness Variation Database, Shearer *et al*.^20^ reported exceptions for alleles of four genes: *GJB2*, *SLC26A4*, *PCDH15*, and *MYO15A*, whereas according to our approach, the only exception is one *GJB2* variant.

Various studies have claimed discovery of deafness-causing genes; therefore, understanding of gene-specific characteristics is important for the interpretation of gene variants associated with NSHL. Tayoun *et al*.^9^ have provided evidence-based approach for analysis of gene-disease associations and their clinical value in hearing loss, and similar algorithms in gene curation for different NSHL aspects should be incorporated in future studies and applied to a rapidly growing gene list. In this study, we adopted several strategies for optimization of the ACMG guidelines for hearing loss; similar attempts toward ACMG guideline refinement have recently been made in various diseases^24–26^. Thus, we incorporated reliable gene evaluation results such as identification of LoF genes and intragenic regions based on gene tolerance to different mutations into the NSHL-optimized ACMG guidelines^27^. Although some researchers prefer uniform application of ACMG guidelines for simplicity and convenience^28^, we believe that our NSHL-specific ACMG guidelines would enhance the efficiency of assessing the pathogenic potential of a gene variant through prompt adaptation of high-quality data by professional curation.

In response to the urgent need for optimization of NSHL variant interpretation, a large panel of experts called ClinGen Hearing Loss Working Group has released their opinion on specification of the ACMG guidelines for genetic hearing loss^13^. Upgraded clarification of original ACMG principles enabled ClinGen Working Group to provide meticulous modifications for hearing loss variants; however, they applied this specified guideline to small number of variants (51 variants in 9 genes). We analyzed NSHL-associated variants reported so far and presented the results of systematic assessments, emphasizing the importance of NSHL-specific interpretation approaches for genetic diagnosis.

Contamination of the gnomAD with pathogenic variants, or HGMD and ClinVar with benign variants is another issue that should be considered in the context of NSHL genetic landscape. Only a few NSHL-related genes have been selected for report even in newborn genomic sequencing^29^, probably because some genetic variants linked to NSHL are partly associated with late onset or moderate penetrance with unclear severity, which may explain why some pathogenic variants have a relatively high AF in gnomAD. Hereditary hearing loss can be further aggravated by such factors as aging and noise exposure^30^, which might lead to confusion and deposition of false-positive data in mutation databases, including HGMD and ClinVar. As the clinical utility of *in silico* algorithms was shown to be insufficient for variant reclassification^31^, our study demonstrated that accurate calculation of AF thresholds might minimize errors and help avoiding false-negative or false-positive results, especially in the identification of benign variants.

Several previous studies have attempted to improve the accuracy in clinical variant interpretation for Mendelian disorders by using large databases^32–34^. In our study, we specifically focused on NSHL, which has a very heterogeneous genetic landscape; therefore, comprehensive assessment should be performed to make appropriate updates as new information emerges. Our approach, together with other strategies such as AUDIOME^10^, a tiered exome sequencing-based panel, might enhance the clinical utility of NGS and promote the implementation of precision medicine in NSHL^35^.

Our study has several limitations. First, our results were obtained based on the global AF and, thus, may be different from those obtained using other population-specific AFs. Indeed, in the context of clinical genetic testing, patient ethnicity should be considered. However, the determination of AF thresholds might require as many allele counts from large populations as possible, and since the pathogenicity of certain variants is not ethnicity-specific, we established universal minor allele frequency cutoff values for hearing loss variants. Second, there is an ongoing debate about the existence of accurate prevalence and penetrance data. Nevertheless, we think that in our study, meaningful calculations of AF thresholds were done based on a statistically robust framework for NSHL variant interpretation. As disease-specific thresholds are recommended, we believe that our safe, although seemingly lenient, AF thresholds should increase cost-effectiveness in NSHL genetic testing.

In conclusion, we suggest AF thresholds for NSHL-linked gene variants using gnomAD-based filtering AFs for precise evaluation of variant pathogenicity in the context of NSHL-optimized ACMG guidelines. This systematic approach can be applied to evaluate causality of sequence variants in hearing loss-related genes, which would promote accurate diagnosis of hearing loss and development of precision medicine approaches clearly beneficial for NSHL patients.

## Supplementary information

Supplementary information
