# Supplementary material for: Systematic evaluation of gene variants linked to hearing loss based on allele frequency threshold and filtering allele frequency
Source: Sci Rep. 2019 Mar 14;9:4583. doi: 10.1038/s41598-019-41068-6 (PMC6418148; doi:10.1038/s41598-019-41068-6)
Supplement: Supplementary file 1 — Supplementary information [file 41598_2019_41068_MOESM1_ESM.pdf]

Supplementary Information

Systematic evaluation of gene variants linked to hearing loss based on allele frequency threshold and filtering allele frequency

John Hoon Rim, Joon Suk Lee, Jinsei Jung, Ji Hyun Lee, Seung-Tae Lee, Jong Rak Choi, Jae Young Choi, Min Goo Lee, Heon Yung Gee

Supplementary Table S1. A list of 97 curated known nonsyndromic hearing loss genes evaluated in this study

#	Gene	OMIM number	Mode	HHL	DVD	Reference 1 (2016)	Reference 2 (2018)_AUDIOME	Reference 3 (2018)_OtoGenome	Detailed description in OMIM (matched phenotype MIM number)
1	*ACTG1*	102560	AD	Y	Y	Evidencial level 3	Y	Y (category 2)	Y (604717)
2	*ADCY1*	103072	AR	Y	Y			Y (category 3)	Y (610154)
3	*AIFM1*	300169	XR	Y	Y				Y (300614)
4	*BSND*	606412	AR	Y	Y	Evidencial level 3	Y	Y (category 1)	Y (602522)
5	*CABP2*	607314	AR	Y	Y		Y	Y (category 3)	Y (614899)
6	*CCDC50*	611051	AD	Y	Y	Evidencial level 2	Y	Y (category 2)	Y (607453)
7	*CD164*	603356	AD	Y	Y			Y (category 3)	Y (616969)
8	*CDC14A*	603504	AR	Y	Y		Y	Y (category 3)	Y (616958)
9	*CDH23*	605516	AR	Y	Y	Evidencial level 3	Y	Y (category 3)	Y (601386)
10	*CEACAM16*	614591	AD	Y	Y	Evidencial level 2	Y	Y (category 1)	Y (614614)
11	*CIB2*	605564	AR	Y	Y	Evidencial level 3	Y	Y (category 3)	Y (609439)
12	*CLDN14*	605608	AR	Y	Y	Evidencial level 3	Y	Y (category 3)	Y (614035)
13	*CLIC5*	607293	AR	Y	Y		Y	Y (category 3)	Y (616042)
14	*COCH*	603196	AD	Y	Y	Evidencial level 3	Y	Y (category 2)	Y (601369)
15	*COL11A2*	120290	AD/AR	Y	Y	Evidencial level 3	Y	Y (category 2)	Y (601868, 609706)
16	*COL4A6*	303631	XR	Y	Y				Y (300914)
17	*CRYM*	123740	AD	Y	Y				Y (616357)
18	*DCDC2*	605755	AR	Y	Y				Y (610212)
19	*DFNA5*	608798	AD	Y	Y	Evidencial level 3	Y	Y (category 2)	Y (600994)
20	*DFNB59*	610219	AR	Y	Y	Evidencial level 3	Y	Y (category 2)	Y (610220)
21	*DIABLO*	605219	AD	Y	Y		Y	Y (category 2)	Y (614152)
22	*DIAPH1*	602121	AD	Y	Y	Evidencial level 2	Y	Y (category 3)	Y (124900)
23	*DMXL2*	612186	AD	Y			Y		Y (617605)
24	*ELMOD3*	615427	AR	Y	Y				Y (615429)
25	*EPS8*	600206	AR	Y	Y		Y	Y (category 1)	Y (615974)
26	*EPS8L2*	614988	AR	Y	Y				Y (617637)
27	*ESPN*	606351	AD/AR	Y	Y	Evidencial level 3	Y	Y (category 1)	Y (609006)
28	*ESRRB*	602167	AR	Y	Y	Evidencial level 3	Y	Y (category 1)	Y (608565)
29	*EYA4*	603550	AD	Y	Y	Evidencial level 3	Y	Y (category 3)	Y (601316)
30	*FAM65B*	611410	AR	Y	Y		Y		Y (616515)
31	*GIPC3*	608792	AR	Y	Y	Evidencial level 3	Y	Y (category 1)	Y (601869)
32	*GJB2*	121011	AD/AR	Y	Y	Evidencial level 3	Y	Y (category 1)	Y (601544, 220290)
33	*GJB3*	603324	AD/AR	Y	Y				Y (612644, 220290)
34	*GJB6*	604418	AD/AR	Y	Y	Evidencial level 3	Y	Y (category 2)	Y (612643, 612645, 220290)
35	*GRHL2*	608576	AD	Y	Y	Evidencial level 2	Y	Y (category 1)	Y (608641)
36	*GRXCR1*	613283	AR	Y	Y	Evidencial level 2	Y	Y (category 1)	Y (613285)
37	*GRXCR2*	615762	AR	Y	Y		Y		Y (615837)
38	*HGF*	142409	AR	Y	Y		Y	Y (category 3)	Y (608265)
39	*HOMER2*	604799	AD	Y	Y		Y		Y (616707)
40	*ILDR1*	609739	AR	Y	Y	Evidencial level 3	Y	Y (category 2)	Y (609646)
41	*KARS*	601421	AR	Y	Y	Evidencial level 2		Y (category 2)	Y (613916)
42	*KCNQ4*	603537	AD	Y	Y	Evidencial level 3	Y	Y (category 2)	Y (600101)
43	*KITLG*	184745	AD	Y	Y			Y (category 2)	Y (616697)
44	*LHFPL5*	609427	AR	Y	Y	Evidencial level 3	Y	Y (category 1)	Y (610265)
45	*LOXHD1*	613072	AR	Y	Y	Evidencial level 3	Y	Y (category 3)	Y (613079)
46	*LRTOMT*	612414	AR	Y	Y	Evidencial level 3	Y	Y (category 3)	Y (611451)
47	*MARVELD2*	610572	AR	Y	Y	Evidencial level 3	Y	Y (category 2)	Y (610153)
48	*MCM2*	116945	AD	Y	Y				Y (616968)
49	*MET*	164860	AR	Y	Y				Y (616705)
50	*MIR96*	611606	AD	Y	Y	Evidencial level 3			Y (613074)
51	*MSRB3*	613719	AR	Y	Y	Evidencial level 2	Y	Y (category 3)	Y (613718)
52	*MYH14*	608568	AD	Y	Y	Evidencial level 2	Y	Y (category 2)	Y (600652)
53	*MYH9*	160775	AD	Y	Y	Evidencial level 3	Y	Y (category 1)	Y (603622)
54	*MYO15A*	602666	AR	Y	Y	Evidencial level 3	Y	Y (category 1)	Y (600316)
55	*MYO3A*	606808	AR	Y	Y	Evidencial level 3	Y	Y (category 1)	Y (607101)
56	*MYO6*	600970	AD/AR	Y	Y	Evidencial level 3	Y	Y (category 2)	Y (606346, 607821)
57	*MYO7A*	276903	AD/AR	Y	Y	Evidencial level 3	Y	Y (category 3)	Y (601317, 600060)
58	*NARS2**	612803	AR	Y	Y				NONE
59	*NLRP3*	606416	AD	Y	Y			Y (category 2)	Y (617772)
60	*OSBPL2*	606731	AD	Y	Y		Y	Y (category 3)	Y (616340)
61	*OTOA*	607038	AR	Y	Y	Evidencial level 3	Y	Y (category 3)	Y (607039)
62	*OTOF*	603681	AR	Y	Y	Evidencial level 3	Y	Y (category 3)	Y (601071)
63	*OTOG*	604487	AR	Y	Y	Evidencial level 2	Y	Y (category 3)	Y (614945)
64	*OTOGL*	614925	AR	Y	Y	Evidencial level 2	Y	Y (category 1)	Y (614944)
65	*P2RX2*	600844	AD	Y	Y	Evidencial level 3	Y	Y (category 3)	Y (608224)
66	*PCDH15*	605514	AR	Y	Y	Evidencial level 3	Y	Y (category 3)	Y (609533)
67	*PNPT1*	610316	AR	Y	Y				Y (614934)
68	*POU3F4*	300039	XR	Y	Y	Evidencial level 3	Y	Y (category 1)	Y (304400)
69	*POU4F3*	602460	AD	Y	Y	Evidencial level 3	Y	Y (category 1)	Y (602459)
70	*PRPS1*	311850	XL	Y	Y	Evidencial level 3	Y	Y (category 2)	Y (304500)
71	*PTPRQ*	603317	AR	Y	Y		Y	Y (category 1)	Y (617663, 613391)
72	*RDX*	179410	AR	Y	Y	Evidencial level 2	Y	Y (category 3)	Y (611022)

73	*ROR1*	602336	AR	Y	Y				Y (617654)
74	*S1PR2*	605111	AR	Y	Y		Y	Y (category 1)	Y (610419)
75	*SERPINB6*	173321	AR	Y	Y	Evidencial level 2	Y	Y (category 3)	Y (613453)
76	*SIX1*	601205	AD	Y	Y	Evidencial level 3	Y	Y (category 1)	Y (605192)
77	*SLC17A8*	607557	AD	Y	Y		Y		Y (605583)
78	*SLC26A4*	605646	AR	Y	Y	Evidencial level 3	Y	Y (category 1)	Y (600791)
79	*SLC26A5*	604943	AR	Y	Y				Y (613865)
80	*SMPX*	300226	XD	Y	Y	Evidencial level 3	Y	Y (category 1)	Y (300066)
81	*STRC*	606440	AR	Y	Y	Evidencial level 3	Y	Y (category 1)	Y (603720)
82	*SYNE4*	615535	AR	Y	Y	Evidencial level 2	Y	Y (category 2)	Y (615540)
83	*TBC1D24*	613577	AD/AR	Y	Y	Evidencial level 3	Y	Y (category 2)	Y (614617, 616044)
84	*TECTA*	602574	AD/AR	Y	Y	Evidencial level 3	Y	Y (category 1)	Y (601543, 603629)
85	*TJP2*	607709	AD	Y	Y				Y
86	*TMC1*	606706	AD/AR	Y	Y	Evidencial level 3	Y	Y (category 1)	Y (606705, 600974)
87	*TMEM132E*	616178	AR	Y	Y				Y
88	*TMIE*	607237	AR	Y	Y	Evidencial level 3	Y	Y (category 1)	Y (600971)
89	*TMPRSS3*	605511	AR	Y	Y	Evidencial level 3	Y	Y (category 3)	Y (601072)
90	*TNC*	187380	AD	Y	Y				Y (615629)
91	*TPRN*	613354	AR	Y	Y	Evidencial level 3	Y	Y (category 1)	Y (613307)
92	*TRIOBP*	609761	AR	Y	Y	Evidencial level 3	Y	Y (category 3)	Y (609823)
93	*TSPEAR*	612920	AR	Y	Y	Evidencial level 2	Y		Y (614861)
94	*USH1C*	605242	AR	Y	Y	Evidencial level 3	Y	Y (category 3)	Y (602092)
95	*WBP2*	606962	AR	Y					Y (617639)
96	*WFS1*	606201	AD	Y	Y	Evidencial level 3	Y	Y (category 2)	Y (600965)
97	*WHRN*	607928	AR	Y	Y	Evidencial level 3	Y	Y (category 3)	Y (607084)

*Multiple reports (PMID: 25807530, 28077841) provide sufficient evidence to be included in the analysis.

Abbreviations: AD, autosomal dominnat; AR, autosomal recessive; XR, X-recessive; XL, X-linked; OMIM, Online Mendelian Inheritance in Man; HHL, Hereditary Hearing Loss database; DVD, Deafness Variation Database; Y, yes.

References: #1, Abou Tayoun et al. Genet Med. 2016;18(6):545-53.; #2, Guan et al. Genet Med. 2018 in press. doi: 10.1038/gim.2018.48.; #3, DiStefano et al. J Mol Diagn. 2018;20(6):789-801.

Supplementary Table S2. Distribution of variants in terms of observed allele frequency using empirical approach of bottom-up analysis

**Allele frequency category**	**AF = 0**	**0< AF <0.005%**	**0.005% ≤ AF <0.01%**	**0.01% ≤ AF <0.05%**	**0.05% ≤ AF <0.1%**	**0.1% ≥ AF**	**Note; sum of lowest categories (AF = 0 and 0< AF <0.005%)**
Database type	**variant number (percentage of variants in the corresponding category within all variants from the database)**						
gnomAD	1,951 (55.0%)	1110 (31.3 %)	143 (4.0 %)	175 (4.9 %)	57 (1.6 %)	113 (3.2 %)	3061 (86.3 %)
ExAC	2323 (65.5%)	733 (20.7 %)	137 (3.9 %)	185 (5.2 %)	59 (1.7 %)	112 (3.2 %)	3056 (86.2 %)
EVS	3115 (87.8%)	0 (0 %)*	171 (4.8 %)	114 (3.2 %)	52 (1.5 %)	97 (2.7 %)	3115 (87.8%)
1000G	3182 (89.7%)	0 (0 %)*	0 (0.0 %)*	190 (5.4 %)	56 (1.6 %)	121 (3.4 %)	3182 (89.7%)

*Variants with AF of 0 < AF < 0.005% according to EVS and variants with AF of 0 < AF < 0.01% according to 1000G were understandably absent due to smaller population sizes of EVS (n=6,503) and 1000G (n = 2,504) compared to those of gnomAD and ExAC, as 1/13006 (0.0077%) and 1/5008 (0.019%) are greater than 0.005% and 0.01%, respectively.
Abbreviations: AF, allele frequency.

Supplementary Table S3. Profiles of 45 variants with observed and filtering allele frequencies above thresholds

Gene Symbol	Nucleotide Change	Amino acid Change	dbSNP147	Filtering gnomAD AF	Filtering ExAC AF	gnomAD AF	ExAC AF	ESP AF	1000G AF	PP2 humvar	SIFT	Condel	CADD	HGMD	ClinVar	NSHL-optimized ACMG class
**DOMINANT GENES**																
KCNQ4	c.1365T>G	p.His455Gln	rs34287852	0.174971	0.200432	0.176500	0.202841	0.175127	0.094050	Benign (0.005)	Tol (0.35)	Neu (0.021)	12.73	DP	B	Benign
WFS1	c.577A>C	p.Lys193Gln	rs41264699	0.003424	0.003759	0.003900	0.004056	0.002230	0.002196	Dam (0.46)	Tol (0.11)	Neu (0.422)	17.83	DM?	B|LB	Benign
WFS1	c.2335G>A	p.Val779Met	rs141328044	0.002158	0.001984	0.001700	0.002205	0.008242	0.006589	Dam (0.685)	Tol (0.15)	Del (0.485)	24	DM?	B|LB	Likely benign
WFS1	c.2611G>A	p.Val871Met	rs71532874	0.007239	0.007530	0.007700	0.007949	0.007304	0.003594	Benign (0.019)	Tol (0.08)	Neu (0.301)	21.5	DM?	B|LB	Benign
SLC17A8	c.1120G>T	p.Ala374Ser	rs138307707	0.001239	0.001082	0.001300	0.001244	0.000000	0.002196	Dam (0.964)	Del (0)	Del (0.851)	29.6	DM	-	Variant of unknown significance
MYH14	c.1150G>T	p.Gly384Cys	rs119103280	0.002817	0.002609	0.003000	0.002858	0.002824	0.002396	Dam (0.977)	Del (0)	Del (0.863)	25.8	DM?	***LB|P***	Likely benign
**RECESSIVE GENES**																
BSND	c.127G>A	p.Val43Ile	rs34561376	0.028916	0.027118	0.026000	0.027903	0.042596	0.081669	Benign (0)	Tol (0.44)	Neu (0.013)	0.045	FP	B|LB	Benign
DFNB59	c.874G>A	p.Gly292Arg	rs79399438	0.027655	0.027976	0.028100	0.028774	0.024145	0.044329	Benign (0.201)	Del_LC (0.01)	Neu (0.425)	23	DM?	B|LB	Likely benign
OTOF	c.3751T>G	p.Cys1251Gly	rs41288773	0.015276	0.016617	0.015800	0.017246	0.014073	0.006789	Benign (0)	Tol (0.58)	Neu (0.005)	0.003	DM?	B	Likely benign
OTOF	c.3470G>A	p.Arg1157Gln	rs56054534	0.007744	0.007639	0.008000	0.008060	0.010995	0.004593	Dam (0.998)	Tol (0.91)	Del (0.474)	26.7	DM?	B	Likely benign
OTOF	c.2464C>T	p.Arg822Trp	rs80356570	0.014115	0.015316	0.014500	0.015965	0.018627	0.006190	Dam (0.619)	Del (0)	Del (0.660)	32	DM?	B	Likely benign
OTOF	c.158C>T	p.Ala53Val	rs1879761	0.060283	0.052644	0.064800	0.053734	0.003614	0.100240	Benign (0.196)	Del (0.04)	Neu (0.367)	23.1	DM?	B|LB	Benign
OTOF	c.145C>T	p.Arg49Trp	rs61746568	0.008731	0.007954	0.009500	0.008381	0.004306	0.009385	Dam (0.998)	Del (0)	Del (0.919)	34	DM	B	Likely benign
SLC22A4	c.-1875T>A	NA	rs3761661	0.073804	NA	0.076300	0.000000	0.000000	0.109425	No	No	No	_	FP	No	Benign
SLC22A4	c.1046+5G>A	NA	rs2304081	0.077799	0.076905	0.078100	0.078221	0.068199	0.113618	No	No	No	12.04	FP	No	Benign
SLC26A5	c.-53-2A>G	NA	rs116900495	0.007382	NA	0.007800	0.000000	0.000000	0.003794	No	No	No	22.3	DM?	B|VUS	Benign
SLC26A4	c.1790T>C	p.Leu597Ser	rs55638457	0.007656	0.007831	0.008400	0.008256	0.006305	0.008586	Dam (0.987)	Del (0)	Del (0.881)	29.3	DM?	B|LB	Benign
SLC26A4	c.1826T>G	p.Val609Gly	rs17154335	0.013665	0.012638	0.010400	0.013176	0.048901	0.045727	Benign (0)	Tol (0.37)	Neu (0.019)	18.12	DM?	LB	Likely benign
PCDH15	c.4409+3011_4409+3013delAAC		rs113363047	0.008865	0.008536	0.007000	0.008978	0.035155	0.031350	No	No	No	8.44	DM?	***B|P***	Likely benign
PCDH15	c.1319A>C	p.Asp440Ala	rs4935502	0.231215	0.235725	0.238400	0.238024	0.131632	0.345248	Dam (0.492)	Del (0.01)	Del (0.570)	24.2	DM	B|LB	Benign
CDH23	c.1096G>A	p.Ala366Thr	rs143282422	0.006843	0.006610	0.007300	0.007002	0.008121	0.001997	No	No	No	25.7	DM?	-	Likely benign
CDH23	c.2568C>G	p.Ile856Met	rs188498736	0.006344	0.004650	0.006500	0.005000	0.000300	0.001198	Dam (0.996)	Del (0)	Del (0.935)	23.5	DM?	B|VUS	Likely benign
CDH23	c.3625A>G	p.Thr1209Ala	rs41281314	0.013415	0.012300	0.010700	0.012834	0.046686	0.049321	No	No	No	23.5	DM?	***VUS|B|LB|P***	Likely benign
CDH23	c.4858G>A	p.Val1620Met	rs41281330	0.016213	0.021911	0.017300	0.022736	0.010616	0.012380	No	No	No	29.1	DM?	B	Likely benign
CDH23	c.5418C>G	p.Asp1806Glu	rs74145660	0.013677	0.013888	0.013900	0.014453	0.012923	0.023962	No	No	No	22.4	DM?	B|LB	Likely benign
CDH23	c.6847G>A	p.Val2283Ile	rs41281334	0.052581	0.048506	0.055200	0.049555	0.033958	0.036741	No	No	No	4.949	R	B|LB	Likely benign
STRC	c.179T>C	p.Phe60Ser	rs2729509	0.337683	0.382607	0.347500	0.449219	0.000000	0.436102	Benign (0)	Tol (1)	Neu (0.000)	0.003	R	B	Benign
STRC	c.52_54del	p.Leu18del	NA	0.183673	NA	0.196600	0.000000	0.000000	0.000000	No	No	No	_	DM?	-	Likely benign
MYO15A	c.1783G>A	p.Ala595Thr	rs2955365	0.445098	0.524637	0.453700	0.528876	0.335434	0.553514	Benign (0.186)	Tol_LC (0.06)	Neu (0.340)	23.1	R	B|LB	Benign
MYO15A	c.2152T>G	p.Trp718Gly	rs2955367	0.418646	0.598398	0.447600	0.611200	0.000000	0.496006	Benign (0.161)	Del_LC (0.02)	Neu (0.398)	11.38	R	B|LB	Benign
MYO15A	c.9478C>T	p.Leu3160Phe	rs140029076	0.006624	0.006482	0.006900	0.006870	0.008152	0.005990	Benign (0.277)	Del (0.04)	Neu (0.378)	22	DM?	B	Likely benign
LOXHD1	c.2825_2827delAGA	p.Lys942del	rs142960762	0.016716	0.025149	0.018200	0.026981	0.021473	0.000000	No	No	No	19.63	DM	B	Likely benign
LOXHD1	c.4526G>A	p.Gly1509Glu	rs187587197	0.008189	0.005257	0.008000	0.006100	0.003500	0.002396	Dam (1)	Del (0)	Del (0.945)	33	DM	B	Likely benign
CLDN14	c.11C>T	p.Thr4Met	rs113831133	0.026741	0.037692	0.024300	0.040294	0.043946	0.047125	Benign (0.049)	Tol (1)	Neu (0.003)	6.269	DM?	B|LB	Likely benign
TMPRSS3	c.617-3_617-2dupTA	NA	rs56283966	0.117429	0.121536	0.119900	0.123196	0.090829	0.173722	No	No	No	23.5	DM?	B|LB	Benign
TMPRSS3	c.268G>A	p.Ala90Thr	rs45598239	0.032002	0.032146	0.031800	0.032999	0.034984	0.010184	Dam (0.816)	Tol (0.22)	Neu (0.350)	22.9	DM?	B	Likely benign
TPRN	c.559G>T	p.Ala187Ser	rs9411313	0.265431	0.000000	0.282200	0.000000	0.000000	0.243810	Benign (0.004)	Tol (0.12)	Neu (0.032)	0.002	DM	B	Benign
TRIOBP	c.1193_1195delAAC	p.Gln398del	COSM5713391	0.389910	0.388755	0.393300	0.391714	0.353190	0.336262	No	No	No	8.833	R	-	Benign
**DOMINANT / RECESSIVE GENES**																
GJB3	c.94C>T	p.Arg32Trp	rs1805063	0.022923	0.023479	0.023700	0.024209	0.023066	0.012979	Dam (1)	Del (0)	Del (0.945)	27.5	DM?	B|LB	Likely benign
TMC1	c.247_249delGAA	p.Glu83del	rs376040866	0.009527	0.010344	0.010000	0.010833	0.045004	0.004393	No	No	No	20.5	DM	B|LB	Likely benign
MYO7A	c.5156A>G	p.Tyr1719Cys	rs77625410	0.020210	0.022249	0.016100	0.023980	0.042405	0.043930	Dam (0.997)	Del (0.04)	Del (0.815)	25.2	DM?	B|LB	Likely benign
GJB2	c.457G>A	p.Val153Ile	rs111033186	0.008316	0.010085	0.009400	0.010566	0.002230	0.013179	Benign (0.005)	Tol (1)	Neu (0.000)	5.981	DM?	LB	Likely benign
GJB2	c.380G>A	p.Arg127His	rs111033196	0.012160	0.014793	0.014200	0.015374	0.002307	0.026957	Benign (0)	Tol (0.13)	Neu (0.253)	23.1	DM?	VUS|B	Likely benign
GJB2	c.341A>G	p.Glu114Gly	rs2274083	0.014175	0.014021	0.014400	0.014587	0.000615	0.030951	Benign (0)	Tol (0.31)	Neu (0.027)	2.639	DFP	B|LB	Likely benign
GJB2	c.109G>A	**p.Val37Ile***	rs72474224	0.007289	0.006208	0.007556	0.006587	0.001307	0.015375	Dam (0.995)	Tol (0.13)	Del (0.710)	10.55	DM	***LP|P***	**Pathogenic**
GJB2	c.101T>C	p.Met34Thr	rs35887622	0.008704	0.008073	0.008700	0.008504	0.010226	0.005990	Benign (0.065)	Tol (0.05)	Neu (0.350)	14.43	DM	***VUS|LB|LP|P***	Variant of unknown significance
GJB2	c.79G>A	p.Val27Ile	rs2274084	0.049691	0.044380	0.053200	0.045381	0.002537	0.071885	Dam (0.998)	Tol (0.12)	Del (0.735)	16.99	DP	B|LB	Benign
GJB2	c.-22-12C>T	NA	rs9578260	0.022854	0.020602	0.017300	0.021288	0.079769	0.074880	No	No	-	5.742	DM?	B	Likely benign

*The red bolded variant is the only exception of pathogenic variant with higher filtering allele frequencies than thresholds.

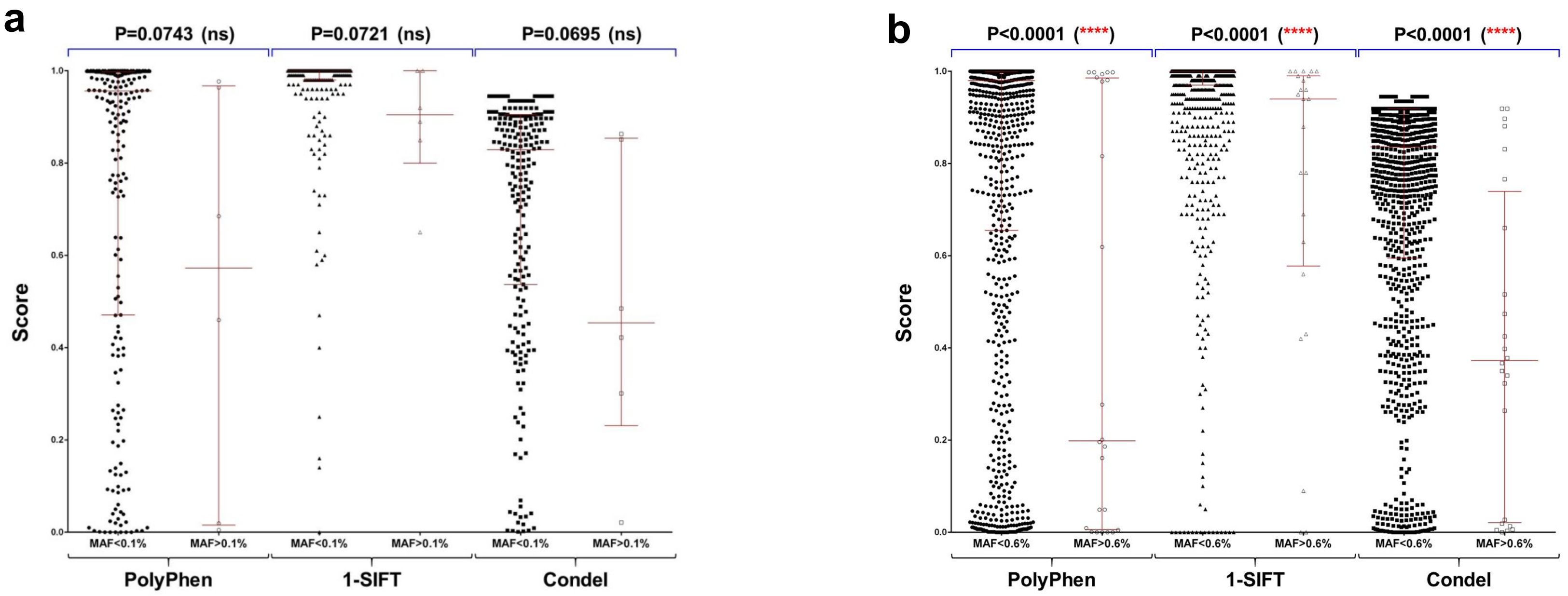

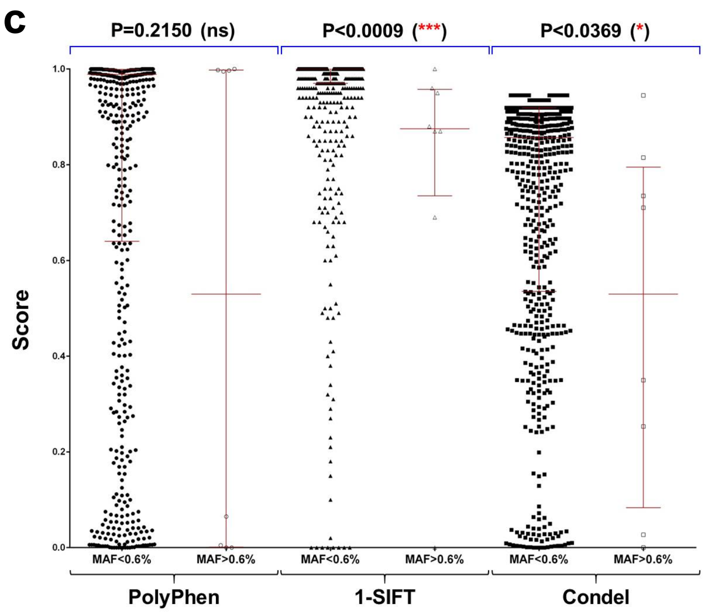

Supplementary Figure S1. Comparative analysis of prediction scores of missense variants according to allele frequency thresholds using PolyPhen-2, SIFT, and Condel. (a) For 28 dominant genes, the predicted scores of 244 missense mutations were not significantly different between variants with AFs below and above the threshold. (b) For 57 recessive genes, prediction scores of 1,040 missense mutations were statistically different between variants with AFs below and above the threshold. (c) For 10 dominant/recessive genes, the prediction scores of 668 missense mutations were significantly different between variants with AFs below and above the threshold. Comparisons were performed using Mann-Whitney tests. Two genes (*CD164* and *EPS8L2*), in which no missense variant linked to hearing loss has been reported so far, were excluded in the analysis. AF, allele frequency; ns, not statistically significant.

SUPPLEMENTARY NOTE 1 – Optimization and adaptation of 2015 ACMG guideline rules to hearing loss genes

In this study, all the 28 original 2015 ACMG guideline rules were reviewed and optimized to apply for hearing loss genes appropriately incorporating well-organized and high-quality evidences published in recent years. As a result, a total of 12 rules were newly established, 17 rules were changed, and 1 rule was neglected. However, scoring system was maintained as the 2015 ACMG guideline as weighting and leveraging each component is still in controversy and might provide sources of biases.

1. Pathogenic

1.1. Very strong

PVS1 – null variant (nonsense, frameshift, canonical ±1 or 2 splice sites, initiation codon, single or multiexon deletion) in a gene where LOF is a known mechanism of disease

→ Whether a specific gene was associated with LOF as mechanism of hearing loss was determined by previous studies as well as pLI score over 0.9. Assignment of LOF gene was conservatively applied and therefore other genes might have chances to be included in the list in the future. Currently, a total of 49 genes were designated as LoF hearing loss genes.

Gene List	Reasons for LOF gene assignment
BSND	Mutational spectrum including LoF, and pLI score over 0.9
CCDC50	Mutational spectrum with only LoF
CDH23	Mutational spectrum including LoF, and pLI score over 0.9
CLDN14	Mutational spectrum including LoF, and pLI score over 0.9
COL11A2	Mutational spectrum including LoF, and pLI score over 0.9
DFNA5	Mutational spectrum with only exon 8 skipping
DFNB59	Mutational spectrum including LoF, and pLI score over 0.9
DIAPH1	Mutational spectrum including LoF, and pLI score over 0.9
ESPN	Mutational spectrum with only LoF
ESRRB	Mutational spectrum including LoF, and pLI score over 0.9

EYA4	Mutational spectrum with only LoF
GIPC3	Mutational spectrum including LoF, and pLI score over 0.9
GJB2	Mutational spectrum including LoF, and pLI score over 0.9
GRHL2	Mutational spectrum with only LoF
GRXCR1	Mutational spectrum including LoF, and pLI score over 0.9
HGF	Mutational spectrum including LoF, and pLI score over 0.9
ILDR1	Mutational spectrum including LoF, and pLI score over 0.9
KCNQ4	Mutational spectrum including LoF, and pLI score over 0.9
LOXHD1	Mutational spectrum including LoF, and pLI score over 0.9
LRTOMT	Mutational spectrum including LoF, and pLI score over 0.9
MARVELD2	Mutational spectrum with only LoF
MSRB3	Mutational spectrum including LoF, and pLI score over 0.9
MYH14	Mutational spectrum including LoF, and pLI score over 0.9
MYH9	Mutational spectrum including LoF, and pLI score over 0.9
MYO15A	Mutational spectrum including LoF, and pLI score over 0.9
MYO3A	Mutational spectrum with only LoF
MYO6	Mutational spectrum including LoF, and pLI score over 0.9
MYO7A	Mutational spectrum including LoF, and pLI score over 0.9
OTOA	Mutational spectrum including LoF, and pLI score over 0.9
OTOF	Mutational spectrum including LoF, and pLI score over 0.9
OTOG	Mutational spectrum including LoF, and pLI score over 0.9
OTOGL	Mutational spectrum with only LoF
PCDH15	Mutational spectrum including LoF, and pLI score over 0.9
POU3F4	Mutational spectrum including LoF, and pLI score over 0.9
POU4F3	Mutational spectrum including LoF, and pLI score over 0.9
RDX	Mutational spectrum including LoF, and pLI score over 0.9
SERPINB6	Mutational spectrum with only LoF
SLC26A4	Mutational spectrum including LoF, and pLI score over 0.9
SMPX	Mutational spectrum with only LoF

STRC	Mutational spectrum including LoF, and pLI score over 0.9
SYNE4	Mutational spectrum with only LoF
TMC1	Mutational spectrum including LoF, and pLI score over 0.9
TMIE	Mutational spectrum including LoF, and pLI score over 0.9
TMPRSS3	Mutational spectrum including LoF, and pLI score over 0.9
TPRN	Mutational spectrum with only LoF
TRIOBP	Mutational spectrum with only LoF
TSPEAR	Mutational spectrum with only LoF
USH1C	Mutational spectrum including LoF, and pLI score over 0.9
WFS1	Mutational spectrum including LoF, and pLI score over 0.9

1.2. Strong

PS1 – Same amino acid change as a previously established pathogenic variant regardless of nucleotide change

→ No change. Definition of established pathogenic variant follows the classification of our modified ACMG guideline for hearing loss genes.

PS2 – De novo (both maternity and paternity confirmed) in a patient with the disease and no family history

→ No change. In this study, we did not consider this criterion unless the parental genotyping was performed in the primary clinical report.

PS3 – Well-established in vitro or in vivo functional studies supportive of a damaging effect on the gene or gene product

→ Level of "damaging effect" was determined by cut-off of 75% compared to normal activity.

If the level of "damaging effect" was less than 50% but did not reach the cut-off of 75%, lowered PS3 or **PM7 (NEW)** was applied.

PS4 – The prevalence of the variant in affected individuals is significantly increased compared with the prevalence in controls

→ As this criterion could be ambiguous and impossible to calculate the true estimates for all hearing loss genes, we applied alternative criterion for hearing loss genes. For the variants with POPMAX AF in gnomAD lower than our thresholds (i.e. 0.1% of AD genes and 0.6% for AR genes), the observed number of reported cases with the same variant in hearing loss patients was counted and different levels of rules were considered according to the number.

1) For the variant with more than 15 hearing loss case reports, **PS5 (NEW)** was applied.

2) For the variant with 6~14 hearing loss case reports, **PM8 (NEW)** was applied.

3) For the variant with 3~5 hearing loss case reports, **PP6 (NEW)** was applied.

1.3. Moderate

PM1 – Located in a mutational hot spot and/or critical and well-established functional domain (e.g., active site of an enzyme) without benign variation

→ Identification of mutational hot spot in hearing loss genes was adopted from a previous study.[1]

Gene List	Domain according to Pfam	Variant type
MYO7A	MyTH4_6052	Missense, in-frame deletion
MYO7A	Outside	Frameshift, nonsense variant, splice site
SLC26A4	Outside	Missense, in-frame deletion

PM2 – Absent from controls (or at extremely low frequency if recessive) in Exome Sequencing Project, 1000 Genomes Project, or Exome Aggregation Consortium

→ Filtering allele frequency from gnomAD/ExAC database was utilized in comparison with our thresholds. ESP and 1000G data were used to check the absolute absence of the variant in the database.

PM3 – For recessive disorders, detected in trans with a pathogenic variant

→ No change. In this study, we did not consider this criterion unless the confirmation of trans status of two variants were thoroughly reviewed.

PM4 – Protein length changes as a result of in-frame deletions/insertions in a nonrepeat region or stop-loss variants

→ No change.

PM5 – Novel missense change at an amino acid residue where a different missense change determined to be pathogenic has been seen before

→ No change. However, this criterion was downgraded to **PP7 (NEW)**, as protein characteristics of remaining 19 amino acids are various in terms of polarity, size, and bond formation.

PM6 – Assumed de novo, but without confirmation of paternity and maternity

→ No change. In this study, we did not consider this criterion unless the parental genotyping was performed in the primary clinical report.

1.4. Supporting

PP1 – Cosegregation with disease in multiple affected family members in a gene definitively known to cause the disease

→ Level of this criterion was upgraded according to corresponding total numbers of segregated affected/unaffected family members in all the reports.

PS6 (NEW) is applied when more than 10 (or 5) affected individuals for AD (or AR) genes were segregated in two or more families.

PM9 (NEW) is applied when more than 6 (or 3) affected individuals for AD (or AR) genes were segregated in two or more families.

PP1 is applied when more than more than 3 (or 2) affected individuals for AD (or AR) genes were segregated in one or more families.

PP2 – Missense variant in a gene that has a low rate of benign missense variation and in which missense variants are a common mechanism of disease

→ Determination of genes with missense variants as the common disease mechanism was adopted from a previous study.

Low rate of benign missense variation was determined by calculating allele frequency sum of missense variants in gnomAD higher than 5%.

Concept of "etiologic fraction" was also adopted from a previous study with cut-off of 0.75.

PP3 – Multiple lines of computational evidence support a deleterious effect on the gene or gene product (conservation, evolutionary, splicing impact, etc.)

→ As increasing number of available in-silico algorithms is limitless, we only apply three most widely used prediction tools (i.e. SIFT, PolyPhen-2, and Condel). If all three prediction tools agree, receive PP3 point.

For all variants which received PP3, conservation across 4 species (Mus musculus, Gallus gallus, Xenopus tropicalis, and Danio rerio) is assessed to additional **PP8 (NEW)** point if all conserved.

PP4 – Patient's phenotype or family history is highly specific for a disease with a single genetic etiology.

→ All variants identified in deaf patients, after alternative causes of hearing loss are excluded, receive PP4 point.

PP5 – Reputable source recently reports variant as pathogenic, but the evidence is not available to the laboratory to perform an independent evaluation

→ Not applicable, since new rules (PS5, PM8 and PP6) is already considered.

2. BENIGN

2.1. Stand-alone

BA1 – Allele frequency is >5% in Exome Sequencing Project, 1000 Genomes Project, or Exome Aggregation Consortium

→ Filtering allele frequency of the variant from gnomAD/ExAC is compared with four-fold of our thresholds (i.e. 0.4% in AD genes and 2.4 %) is used.

Cut-off of 5% is applied for specific ethnic subpopulation. For example, the National Biobank of Korea (NBK) data for Korean population was searched for variant with allele frequency over 5 %.

2.2. Strong

BS1 – Allele frequency is greater than expected for disorder

→ Filtering allele frequency of the variant from gnomAD is compared with our thresholds (i.e. 0.1% in AD genes and 0.6 %) is used.

BS2 – Observed in a healthy adult individual for a recessive (homozygous), dominant (heterozygous), or X-linked (hemizygous) disorder, with full penetrance expected at an early age

→ As penetrance and age of onset could be gene-dependent features, identification of genes with high penetrance AND early onset is defined according to the previous report.[2]

Gene list for BS2 application (n=46): *ACTG1, AIFM1, BSND, CDH23, CLDN14, COCH, COL11A2, DFNA5, DFNB59, ESRRB, EYA4, GIPC3, GJB2, GJB2, ILDR1, KCNQ4, LHFPL5, LOXHD1, LRTOMT, MARVELD2, MYH14, MYH9, MYO15A, MYO3A, MYO6, MYO7A, OTOA, OTOF, OTOGL, PCDH15, POU3F4, POU4F3, PRPS1, SIX1, SLC26A4, SMPX, STRC, TBC1D24, TECTA, TJP2, TMC1, TMIE, TMPRSS3, TRIOBP, USH1C, WFS1*

Similar approach is applied for genes with intermediate penetrance OR late onset with new point **BP8 (NEW)**.

Gene list for BP8 application (n=8): *CCDC50, DIABLO, GRHL2, HGF, KARS, SERPINB6, SYNE4, TSPEAR*

BS3 – Well-established in vitro or in vivo functional studies show no damaging effect on protein function or splicing

→ Level of “no damaging effect” was determined by cut-off of 25% compared to normal activity.

If the level of “damaging effect” was more than 25% but did not reach the cut-off of 50%, lowered BS3 or **BP9 (NEW)** was applied.

BS4 – Lack of segregation in affected members of a family

→ Maximum allowable very major error (i.e. unaffected family member with the corresponding variant) is less than 1. Maximum allowable major error (i.e. affected family member without the corresponding variant) is less than 2.

If the total number of very major error/major error in all reports is observed more than the cut-off thresholds, receive BS4.

BS5 (NEW) – Located in a mutational "cold" spot

→ As a benign component matching with PM1 (Located in a mutational hot spot), we used results from a previous study identifying mutational "cold" spot, which represents tolerant regions to variants, in hearing loss genes.[1] BS5 is activated when missense variant is observed in corresponding cold spots.

Gene List	Domain according to Pfam	Variant type
CDH23	Cadherin_36780/Outside	Missense, in-frame deletion
OTOF	Outside	Frameshift, nonsense variant, splice site
PCDH15	Cadherin_37570/Outside	All variants
MYO15A	Outside	Missense, in-frame deletion
MYO3A	Outside	Missense, in-frame deletion
TRIOBP	Outside	Missense, in-frame deletion
WHRN	Outside	Missense, in-frame deletion

2.3. Supporting

BP1 – Missense variant in a gene for which primarily truncating variants are known to cause disease

→ Only applied for following 14 genes.

1) *DFNA5* (Exon 8 skipping is the only known pathomechanism for the hearing loss in this gene.)

2) *CCDC50, ESPN, EYA4, GRHL2, MARVELD2, MYO3A, OTOGL, SERPINB6, SMPX, SYNE4, TPRN, TRIOBP, TSPEAR* (Mutational spectrum is only known to be LoF in these genes.)

BP2 – Observed in trans with a pathogenic variant for a fully penetrant dominant gene/disorder or observed in cis with a pathogenic variant in any inheritance pattern

→ No change. In this study, we did not consider this criterion unless the confirmation of cis status of two variants was thoroughly reviewed.

BP3 – In-frame deletions/insertions in a repetitive region without a known function

→ Only applied for any in-frame insertion or deletion that falls within a region annotated by repeat masker.

BP4 – Multiple lines of computational evidence suggest no impact on gene or gene product (conservation, evolutionary, splicing impact, etc.)

→ As increasing number of available in-silico algorithms is limitless, we only apply three most widely used prediction tools (i.e. SIFT, PolyPhen-2, and Condel). If all three prediction tools agree, receive PP3 point.

For all variants which received BP4, lack of conservation across 4 species (Mus musculus, Gallus gallus, Xenopus tropicalis, and Danio rerio) is assessed to additional **BP10 (NEW)** point if at least one species has the same amino acid change.

BP5 – Variant found in a case with an alternate molecular basis for disease

→ No change. In this study, we did not consider this criterion.

BP6 – Reputable source recently reports variant as benign, but the evidence is not available to the laboratory to perform an independent evaluation

→ No change. In this study, we did not consider this criterion.

BP7 – A synonymous (silent) variant for which splicing prediction algorithms predict no impact to the splice consensus sequence nor the creation of a new splice site AND the nucleotide is not highly conserved

→ No change. In this study, we did not consider this criterion.

SUPPLEMENTARY NOTE 2 – Rationale for theoretical calculations for allele frequency thresholds of nonsyndromic hearing loss genes

There is no single mutation representing the majority of dominant NSHL genes in a given population.[3] Therefore, to set the AF threshold of the dominant genes, we assumed that the frequency of a single allele including a certain variant causing NSHL is not higher than the prevalence of total hearing loss. As a result, an AF threshold of 0.1% was obtained through Hardy-Weinberg equilibrium.

In the case of recessive genes, we used the following proposed formula suggested by Whiffin et al.[4] maximum credible population AF = √(prevalence) × maximum allelic contribution × √ (maximum genetic contribution) × 1/√(penetrance). Based on the results of a large-scale genetic study of NSHL including up to 1,119 individuals,[5] we can assume that no newly identified variant will be more common. In the present cohort, 21.59% (95/440) of all NSHL patients with known genes had disease-causing variants in GJB2, and the c.35delG (p.Gly12Valfs*2) variant is estimated to account for 37.89% (72/190) of variant GJB2 alleles. Finally, we assumed a penetrance of 1 as the phenotype of recessive genes has nearly 100% penetrance. Therefore, the maximum AF threshold for this allele was $\sqrt{(0.002 \times 0.8 \times 0.7)} \times 0.3789 \times \sqrt{0.2159} \times 1 \leq 0.6\%$

SUPPLEMENTARY NOTE References

1. Amr SS, Al Turki SH, Lebo M, Sarmady M, Rehm HL, Abou Tayoun AN. Using large sequencing data sets to refine intragenic disease regions and prioritize clinical variant interpretation. *Genet Med* 2017; 19(5):496-504.
2. Ceyhan-Birsoy O, Machini K, Lebo MS et al. A curated gene list for reporting results of newborn genomic sequencing. *Genet Med* 2017; 19(7):809-818.
3. Shearer AE, Eppsteiner RW, Booth KT et al. Utilizing ethnic-specific differences in minor allele frequency to recategorize reported pathogenic deafness variants. *Am J Hum Genet* 2014; 95(4):445-453.
4. Whiffin N, Minikel E, Walsh R et al. Using high-resolution variant frequencies to empower clinical genome interpretation. *Genet Med* 2017; 19(10):1151-1158.
5. Sloan-Heggen CM, Bierer AO, Shearer AE et al. Comprehensive genetic testing in the clinical evaluation of 1119 patients with hearing loss. *Hum Genet* 2016; 135(4):441-450.
